# 3D-Printed silicone anatomic patient simulator to enhance training on cardiopulmonary bypass

**DOI:** 10.1051/ject/2023005

**Published:** 2023-06-28

**Authors:** Branden Tyler Messarra, Yaxin Wang, P. Alex Smith, Preston Peak, Deborah L. Adams, Terry N. Crane

**Affiliations:** 1 School of Perfusion Technology, Texas Heart Institute Houston TX 77030 USA; 2 Innovative Device and Engineering Applications (IDEA) Laboratory, Texas Heart Institute Houston TX 77030 USA

**Keywords:** Perfusion, High-fidelity simulation training, Cardiopulmonary bypass, Cardioplegia

## Abstract

*Background*: Simulator training is important for teaching perfusion students fundamental skills associated with CBP before they start working in the clinic. Currently available high-fidelity simulators lack anatomic features that would help students visually understand the connection between hemodynamic parameters and anatomic structure. Therefore, a 3D-printed silicone cardiovascular system was developed at our institution. This study aimed to determine whether using this anatomic perfusion simulator instead of a traditional “bucket” simulator would better improve perfusion students’ understanding of cannulation sites, blood flow, and anatomy. *Methods*: Sixteen students were tested to establish their baseline knowledge. They were randomly divided into two groups to witness a simulated bypass pump run on one of two simulators – anatomic or bucket – then retested. To better analyze the data, we defined “true learning” as characterized by an incorrect answer on the pre-simulation assessment being corrected on the post-simulation assessment. *Results*: The group that witnessed the simulated pump run on the anatomic simulator showed a larger increase in mean test score, more instances of true learning, and a larger gain in the acuity confidence interval. *Conclusions*: Despite the small sample size, the results suggest that the anatomic simulator is a valuable instrument for teaching new perfusion students.

## Overview

Cardiopulmonary bypass (CPB) is a complicated process required for many cardiac surgical procedures, and entry-level perfusionists require proper training and evaluation programs to develop their skills [1]. At teaching institutions, perfusion students practice under the direction of a certified cardiopulmonary perfusionist, and students are trained through a combination of classroom lectures, operating room experience, and hands-on simulator experience [2].

**Figure 1:**
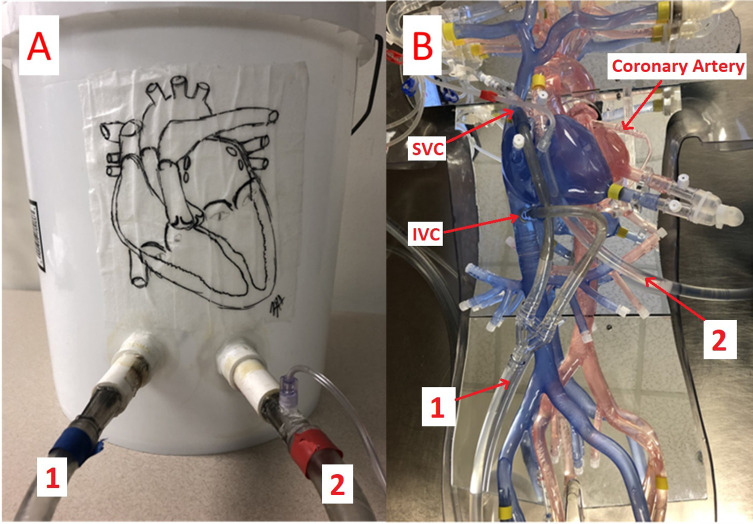
Traditional bucket patient simulator (A) and 3-D printed anatomic patient simulator (B). 1 indicates the venous line; 2 indicates the arterial line. IVC, inferior vena cava; SVC, superior vena cava.

In 1975, a perfusion simulator (Figure 1A) consisting of a 30-gallon tank with tubing to simulate an arterial perfusion line, coronary perfusion line, venous outlet, and suction outlet was developed to instruct perfusion students in the basics of CPB [3]. Simulator experience is important because it helps students acquire fundamental skills associated with CBP and learn to handle different hemodynamic situations before they begin working in the clinic.

Several modern educational perfusion simulators have been developed in recent years and are currently in use [4–7]. High-fidelity simulators have been developed that replicate physiologic parameters and flow dynamics to teach students outside the operating room [8]. However, such devices simulate the different hemodynamic states of the human cardiovascular system in a “black box” that provides no detailed anatomic information. Therefore, it is difficult for students to visually understand the connection between hemodynamic parameters and the anatomic structure of the cardiovascular system. To determine whether an anatomic simulator that mimics human vasculature can improve students’ understanding of CPB-associated human anatomy and flow characteristics, we compared educational outcomes between perfusion students trained with a 3D-printed anatomic simulator (Figure 1A) developed by Texas Heart Institute and students trained with a standard “bucket” simulator (Figure 1B).

The structure of the standard bucket simulator (Figure 1A) is very simple. Only two ports protrude from the bucket: the flow inlet (connected to the venous cannula) and the outlet (connected to the aortic cannula). A connector with a three-way stopcock is located on the arterial line tubing to simulate antegrade cardioplegia delivery or aortic root venting; however, no cardioplegia or retrograde port is available on the bucket.

**Figure 2:**
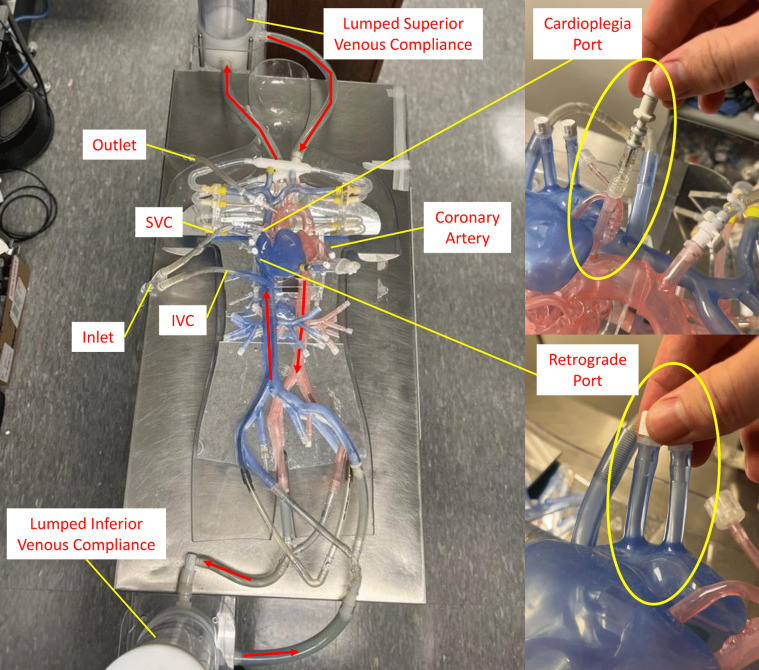
Detailed structure of the 3-D printed anatomic patient simulator. The red arrows indicate the direction of flow. IVC, inferior vena cava; SVC, superior vena cava.

Figure 2 shows in detail the structure of the 3D printed anatomic simulator with two different flow circuits. In the first circuit, the flow enters the inferior and superior vena cava via an inlet venous cannula and exits from the silicone port on the aortic arch via an outlet aortic cannula. In addition, the 3D anatomic model has options for femoral venous and arterial cannulation, as well as visceral and renal ports for simulation of perfusion during left heart bypass. The second circuit uses the antegrade cardioplegia port located at the aortic root and the two retrograde ports located at the right atrium. Thus, the anatomic model can be used to simulate all the functions of CPB.

To simulate the hemodynamics of the human cardiovascular system, two compliance chambers were connected between the arteries and the veins at the “head” and “feet” of the silicone model to simulate capillary compliance. Ports at the femoral arteries and veins were reserved for the connection of a continuous-flow pump (CentriMag) to simulate the flow generated by the heart. The fluid used in this study was 40% glycerin mixed with water, which approximates the viscosity of human blood.

In this study, 16 perfusion students were randomly divided into two groups. They were tested with 5 questions (see Appendix) related to the CPB pre-training course. Then they witnessed a simulated bypass pump run on 1 of the 2 simulators – anatomic or bucket – followed by an immediate retest. To better analyze the data, “true learning” was introduced in the post-data analysis to evaluate the student’s ability to learn after using the simulation models.

## Description

Sixteen new perfusion students who had classroom lectures but no hands-on training were tested to determine their baseline knowledge. The test consisted of five multiple-choice questions relating to cannulation, anatomy, and cardioplegia (see Appendix). In addition, for each question, students rated their confidence in their chosen answer.

The students were then randomly assigned to observe a simulated coronary artery bypass grafting procedure with either a bucket simulator or the anatomic simulator. A staff perfusionist played the role of surgeon, and a senior perfusion student played the role of the perfusionist. The mock surgeon described the desired disposables and how they would be used during the procedure. The mock perfusionist rolled up the arterial line, which the surgeon de-aired and connected to the arterial cannula. Venous and cardioplegia connections were made, and CPB was initiated. The mock surgeon described the operation while making various requests of the mock perfusionist (e.g., pump down, fill the heart, cardioplegia) before weaning and termination of CPB. No adverse events or complications were included in the simulation. During the procedure, the observing students were free to move around and ask questions of the mock perfusionist and the mock surgeon. After the demonstration, the students were re-tested to evaluate for improvement in their knowledge and confidence.

An acuity confidence interval (ACI) was calculated for each student. To do so, we first modified the confidence ratings as follows: If the question was answered correctly, the student’s confidence rating for that question was multiplied by +1; if the question was answered incorrectly, the rating was multiplied by −1. The ACI was then calculated as the sum of the modified confidence scores for each student on each assessment.

## Results

The baseline score for the bucket group was 91%, while that of the anatomic group was 69% (Table 1). After members of each group observed a simulated case, mean test scores increased by 3% in the bucket group and by 18% in the anatomic group. However, because the mean baseline score (i.e., pre-simulation assessment score) was much higher for the bucket group than for the anatomic group, the greater increase in the anatomic group’s mean score between pre- and post-simulation assessment could be explained by a “ceiling effect,” in that the bucket group had little room for improvement.

**Table 1:** Pre- and post-simulation test scores for students in the bucket and anatomic groups.

Bucket group			Anatomic group		
Student	Pre-simulation (%)	Post-simulation (%)	Student	Pre-simulation (%)	Post-simulation (%)
1	100	100	1	100	100
2	100	100	2	100	100
3	60	60	3	60	80
4	80	100	4	40	80
5	100	100	5	60	80
6	100	100	6	20	60
7	100	100	7	100	100
			8	80	100
			9	60	80
Mean	91	94	Mean	69	87

For a more useful analysis, we focused on the interaction between response accuracy and confidence level by analyzing the ACI data in conjunction with the participant’s responses. In doing so, we classified ACIs and pre-and post-simulation responses into three categories (Table 2): true learning, confirmation of a correct answer, and reinforcement of an incorrect answer. True learning (highlighted green in the table) is characterized by an incorrect answer on the pre-simulation assessment being corrected on the post-simulation assessment. Confirmation of a correct answer (highlighted in yellow) is characterized by a student answering a question correctly on both assessments, with an increase in confidence in the post-simulation assessment. Reinforcement of an incorrect answer (highlighted in red) is characterized by an increase in confidence in the post-simulation assessment despite an incorrect answer having been chosen on both assessments.

**Table 2:** Pre- and post-simulation questionnaire scores and the confidence score associated with each question.

		Pre Simulation		Post Simulation		Pre Simulation		Post Simulation		Pre Simulation		Post Simulation		Pre Simulation		Post Simulation		Pre Simulation		Post Simulation	
Group	Student	Q1	C1	Q1	C1	Q2	C2	Q2	C2	Q3	C3	Q3	C3	Q4	C4	Q4	C4	Q5	C5	Q5	C5
Bucket Simulation	1	Correct	3	Correct	5	Correct	4	Correct	5	Correct	5	Correct	5	Correct	4	Correct	4	Correct	4	Correct	5
	2	Correct	5	Correct	5	Correct	5	Correct	5	Correct	5	Correct	5	Correct	5	Correct	5	Correct	5	Correct	5
	3	X	2	X	3	X	2	X	3	Correct	2	Correct	3	Correct	2	Correct	3	Correct	3	Correct	3
	4	X	3	Correct	5	Correct	4	Correct	5	Correct	2	Correct	4	Correct	2	Correct	4	Correct	2	Correct	3
	5	Correct	4	Correct	4	Correct	5	Correct	5	Correct	5	Correct	5	Correct	5	Correct	5	Correct	5	Correct	5
	6	Correct	5	Correct	5	Correct	5	Correct	5	Correct	5	Correct	5	Correct	4	Correct	5	Correct	4	Correct	5
	7	Correct	4	Correct	5	Correct	4	Correct	5	Correct	4	Correct	5	Correct	4	Correct	5	Correct	4	Correct	5

Anatomical Simulation	1	Correct	5	Correct	5	Correct	5	Correct	5	Correct	5	Correct	5	Correct	5	Correct	5	Correct	5	Correct	5
	2	Correct	4	Correct	5	Correct	4	Correct	5	Correct	5	Correct	5	Correct	4	Correct	5	Correct	3	Correct	5
	3	Correct	2	Correct	5	X	2	X	5	Correct	1	Correct	2	X	1	Correct	3	Correct	1	Correct	4
	4	Correct	1	Correct	5	X	2	Correct	3	Correct	2	Correct	5	X	1	Correct	4	X	1	X	3
	5	Correct	5	Correct	5	X	5	X	4	Correct	5	Correct	5	Correct	4	Correct	5	X	4	Correct	5
	6	X	3	Correct	5	Correct	5	Correct	5	X	3	X	5	X	3	Correct	5	X	3	X	5
	7	Correct	5	Correct	5	Correct	5	Correct	5	Correct	5	Correct	4	Correct	5	Correct	5	Correct	5	Correct	5
	8	Correct	5	Correct	5	Correct	5	Correct	5	Correct	5	Correct	5	Correct	5	Correct	5	X	4	Correct	5
	9	X	3	Correct	5	X	5	X	5	Correct	5	Correct	5	Correct	5	Correct	5	Correct	5	Correct	5

Green highlighting indicates true learning, in which an incorrect answer on the pre-simulation assessment was corrected on the post-simulation assessment. Yellow highlighting indicates confirmation of a correct answer, in which a student who answered a question correctly on both assessments had an increased confidence score in the post-simulation assessment. Red highlighting indicates reinforcement of an incorrect answer, in which the student had an increased confidence score in the post-simulation assessment despite having given an incorrect answer on both assessments. C, confidence; Q, question; X, wrong answer.

**Table 3:** Summary of the ACI values in the pre-and post-simulation assessments in the bucket and anatomic groups.

Pre-simulation assessment							Post-simulation assessment							
	Q1	Q2	Q3	Q4	Q5	ACI		Q1	Q2	Q3	Q4	Q5	ACI	Gain in ACI
Bucket														
Student 1	3	4	5	4	4	20	Student 1	5	5	5	4	5	24	4
Student 2	5	5	5	5	5	25	Student 2	5	5	5	5	5	25	0
Student 3	−2	−2	2	2	3	3	Student 3	−3	−3	3	3	3	3	0
Student 4	−3	4	2	2	2	7	Student 4	5	5	4	4	3	21	14
Student 5	4	5	5	5	5	24	Student 5	4	5	5	5	5	24	0
Student 6	5	5	5	4	4	23	Student 6	5	5	5	5	5	25	2
Student 7	4	4	4	4	4	20	Student 7	5	5	5	5	5	25	5
				Mean		17					Mean		21	
Anatomic														
Student 1	5	5	5	5	5	25	Student 1	5	5	5	5	5	25	0
Student 2	4	4	5	4	3	20	Student 2	5	5	5	5	5	25	5
Student 3	2	−2	1	−1	1	1	Student 3	5	−5	2	3	4	9	8
Student 4	1	−2	2	−1	−1	−1	Student 4	5	3	5	4	−3	14	15
Student 5	5	−5	5	4	−4	5	Student 5	5	−4	5	5	5	16	11
Student 6	−3	5	−3	5	5	9	Student 6	5	5	−5	5	5	15	6
Student 7	5	5	5	5	5	25	Student 7	5	5	4	5	5	24	−1
Student 8	5	5	5	5	−4	16	Student 8	5	5	5	5	5	25	9
Student 9	−3	−5	5	5	5	7	Student 9	5	−5	5	5	5	15	8
				Mean		12					Mean		19	

ACI, acuity confidence interval; Q, question.

The anatomic group outperformed the bucket group, having 8 instances of true learning, while the bucket group had only 1. Regarding confirmation of a correct answer, the bucket group had 16 instances, and the anatomic group had 10 instances. The bucket group had 2 instances of reinforcement of an incorrect answer, while the anatomic group had 4 instances.

For the bucket group, the mean ACI was 17 in the pre-simulation assessment and 21 in the post-simulation assessment, for a mean ACI gain of 4. For the anatomic group, the mean ACI was 12 in the pre-simulation assessment and 19 in the post-simulation assessment, for a mean ACI gain of 7.

Considering the ACI of individual students, each group had 1 student (Student 2 in the bucket group and Student 1 in the anatomic group) who achieved a perfect ACI of 25 in both their pre- and post-simulation assessments, thus having no net ACI gain. Every other student in the anatomic group but one (Student 7) had a net ACI gain, and all but 2 other students in the bucket group (Students 3 and 5) had a net ACI gain. The student with the largest gain in ACI was in the anatomic group (+15 for Student 4).

## Discussion

Although the students were randomly assigned to groups, there was a substantial between-groups difference in baseline scores: 91% for the bucket group versus 69% for the anatomic group. This disparity was not the result of differences in the material covered, as all students attended the same lectures. However, students’ backgrounds before perfusion school were different (although none of them had received any instruction in CPB operation before taking the pre-test). Therefore, previous medical experience may have resulted in a difference in baseline scores. Additionally, the sample size was small (only 16 students), so any individual who overperformed or underperformed on the test would have significantly affected their group’s baseline score. However, a substantial increase was observed in student performance on the post-training test.

With a sample size of only 16 students and uneven group sizes, it is difficult to draw any statistically meaningful conclusions from our data. However, the results suggest that an anatomic simulator is a valuable teaching tool with the potential to provide perfusionists with training previously only possible in a clinical setting.

In the current anatomical simulator, the fluid can be driven by a continuous centrifugal pump to simulate the effects of the CPB weaning procedure on the heart. Our ultimate goal is to make sure the model can replicate human hemodynamics, which will require a pulsatile-flow pump instead of a continuous-flow pump to simulate the heart, and the addition of adjustable lumped superior and inferior compliance chambers and resistance at the peripheral locations. Currently, our engineering team is developing a pulsatile-flow pump that can replicate the left ventricle [9, 10]. The next step will be integrating the pulsatile pump into this 3D anatomical model to simulate the heart, so the model can simulate not only the physiological effects of replacing the heart with CPB but also the effects of the CPB weaning procedure. In future work, an anatomic simulator could be used to simulate CPB complications, such as air emboli or CPB component failure during surgery. The anatomic simulator could be used independently or in conjunction with a high-fidelity simulator to produce new perfusionists who are well-prepared for clinical cases.

## Data Availability

The Baseline Assesment used in this study is available in the Appendix of this article.
